# Therapeutic Potential of the Cyclin-Dependent Kinase Inhibitor Flavopiridol on c-Myc Overexpressing Esophageal Cancer

**DOI:** 10.3389/fphar.2021.746385

**Published:** 2021-09-21

**Authors:** Md Sazzad Hassan, Nicholas Cwidak, Chloe Johnson, Silvio Däster, Serenella Eppenberger-Castori, Niranjan Awasthi, Jun Li, Margaret A. Schwarz, Urs von Holzen

**Affiliations:** ^1^ Department of Surgery, Indiana University School of Medicine, South Bend, IN, United States; ^2^ Harper Cancer Research Institute, South Bend, IN, United States; ^3^ University of Notre Dame, Notre Dame, IN, United States; ^4^ University of Basel, Basel, Switzerland; ^5^ Department of Pediatrics, Indiana University School of Medicine, South Bend, IN, United States; ^6^ Goshen Center for Cancer Care, Goshen, IN, United States

**Keywords:** esophageal cancer, c-Myc, flavopiridol, nanoparticle albumin-bound paclitaxel, BMS-754807

## Abstract

Tumors with elevated c-Myc expression often exhibit a highly aggressive phenotype, and c-Myc amplification has been shown to be frequent in esophageal cancer. Emerging data suggests that synthetic lethal interactions between c-Myc pathway activation and small molecules inhibition involved in cell cycle signaling can be therapeutically exploited to preferentially kill tumor cells. We therefore investigated whether exploiting elevated c-Myc expression is effective in treating esophageal cancer with the CDK inhibitor flavopiridol. We found frequent overexpression of c-Myc in human esophageal cancer cell lines and tissues. c-Myc overexpression correlated with accelerated esophageal cancer subcutaneous xenograft tumor growth. Esophageal cancer cells with elevated c-Myc expression were found preferentially more sensitive to induction of apoptosis by the CDK inhibition flavopiridol compared to esophageal cancer cells with lower c-Myc expression. In addition, we observed that flavopiridol alone or in combination with the chemotherapeutic agent nanoparticle albumin-bound paclitaxel (NPT) or in combinations with the targeted agent BMS-754807 significantly inhibited esophageal cancer cell proliferation and subcutaneous xenograft tumor growth while significantly enhancing overall mice survival. These results indicate that aggressive esophageal cancer cells with elevated c-Myc expression are sensitive to the CDK inhibitor flavopiridol, and that flavopiridol alone or in combination can be a potential therapy for c-Myc overexpressing esophageal cancer.

## Introduction

Esophageal cancer (EC) has two main subtypes–esophageal squamous cell-carcinoma (ESCC) and esophageal adenocarcinoma (Pandilla et al., 2013). Although squamous cell-carcinoma accounts for about 90 percent of all cases of esophageal cancer worldwide, esophageal adenocarcinoma (EAC) has become the dominant type of esophageal cancer in the United States (Chai and Jamal, 2012; Rustgi and El-Serag, 2014; Rubenstein and Shaheen, 2015; Torre et al., 2015). Esophageal cancer is one of the most aggressive human cancers with poor prognosis, and the overall 5-years survival rate is less than 20 percent (Zhang, 2013; Domper arnal et al., 2015). Prognosis for esophageal cancer remains poor even with modern combination therapies due to high resistance to chemotherapy (Mawhinney and Glasgow, 2012; Bollschweiler et al., 2015). Although esophageal cancer responds initially to conventional chemotherapy, the clinical benefit is limited, and most patients eventually die from distant metastatic disease (Siegel et al., 2015). Therefore, new therapeutic approaches for treatment improvements are urgently needed.

The c-Myc oncogene is present on chromosome 8q24, and encodes a master transcription factor (Huppi et al., 2012). c-Myc plays a vital role in regulation of cell proliferation, apoptosis, and metabolism. (Russo et al., 2003; Dang, 2013; Mcmahon, 2014; Li E. et al., 2017). Dysregulation of c-Myc was observed in many human cancers (Kalkat et al., 2017). c-Myc amplification has also been shown to be frequent in esophageal adenocarcinoma (von rahden et al., 2006). Some studies found that the c-Myc protein was upregulated in 50% of Barrett’s metaplasia and 90% of esophageal adenocarcinoma. (Tselepis et al., 2003). Thus, regulation of c-Myc is considered a potentially important and effective therapeutic target in the treatment of human cancer. However, despite being an obvious target, inhibiting c-Myc therapeutically has proved to be challenging, and practicable inhibition of this protein with pharmaceuticals has yet to be achieved. Recently, better understanding of its expression and function has led to new therapeutic opportunities.

The promise of molecular targeted therapy for esophageal cancer is to provide selective killing of tumor cells. It requires defined activated oncogenic pathways in the tumor cells, so that selective inhibitors can be found to abrogate these pathways. Emerging data suggests that synthetic lethal interactions between oncogene activation and small molecules involved in cell cycle signaling can be therapeutically exploited to preferentially kill tumor cells (Goga et al., 2007; Yang et al., 2010; Horiuchi et al., 2012; Stine et al., 2015). A potential approach to target c-Myc is to exploit this synthetic lethal interaction between overexpression of c-Myc and inhibition of cyclin-dependent kinases (CDKs) (Goga et al., 2007; Horiuchi et al., 2012). Here we examined the utility of the small molecule CDK inhibitor flavopiridol and the insulin-like growth factor 1 receptor/insulin receptor (IGF-1R/IR) targeted agent BMS-754807 in the treatment of esophageal cancer with elevated expression of c-Myc.

## Materials and Methods

### Cell Lines Culture and Reagents

Human esophageal cancer cell lines (OE19, OE33, ESO26, KYSE270, SK-GT-2, Flo-1, ESO51, OE21, and OACM5.1C) were obtained from Sigma Aldrich (St. Lois, MO) and cultured according to manufacturer’s instructions. Flavopiridol was obtained from Cayman Chemical (Ann Arbor, MI), nanoparticle albumin-bound paclitaxel from Goshen Center for Cancer Care (Goshen, IN), BMS-754807 from Active Biochemical Limited (Maplewood, NJ), the cell proliferation reagent WST-1 from Roche Diagnostic Corporation (Indianapolis, IN), c-Myc siRNA (sc-29226) and control siRNA (sc-37007) from Santa Cruz Biotechnology (Santa Cruz, CA). pcDNA3-cMyc was a gift from Wafik El-Deiry (Addgene plasmid #16011) (Ricci et al., 2004) and pcDNA3-EGFP as a negative control (Addgene plasmid #13031) was purchased.

### Tissue Microarray Construction and Data Collection

Tissue microarray (TMA) blocks of primary esophageal and paired non-malignant adjacent tissue specimens were constructed by using TMA-Grand Master^®^ (3DHisteck, Sysmex AG, Switzerland) (Muenst et al., 2014). We used TMAs containing 1 mm cores of 77 esophageal cancer (26 adenocarcinoma and 51 squamous cell carcinomas) with 25 non-malignant mucosa samples. All specimens were part of the Biobank at the Institute of Pathology, University Hospital Basel, Switzerland. For TMA construction, formalin-fixed, paraffin-embedded tissue blocks were prepared according to standard protocols (Bubendorf et al., 2001).

### Immunohistochemistry

All analyses have been performed on Ventana BenchMark Ultra. Primary antibody used was specific for c-Myc (Cell Signaling #13987, dilution 1:50, Cell Signaling Technology, Danvers, MA). For immunohistochemical staining, 4-μm sections of the TMA blocks were incubated for 30 min with a prediluted rabbit-anti-human c-Myc antibody after heat-induced antigen retrieval with Cell Conditioning Solution (Ventana Medical Systems, Tuscon, AZ, United States). Standard DAB-technique (Optiview DAB IHC Detection Kit, Ventana Medical Systems, Tuscan, AZ, United States) was employed for immunostaining. Immunohistochemistry (IHC) evaluation was performed by a senior consultant pathologist. Frequency and staining intensity of c-Myc by tumor cells were analyzed, and c-Myc expression was quantified using the modified Histo-score (protein score) (Mccarty et al., 1985) with a range of possible scores from 0 to 100. c-Myc expression was dichotomized into two groups according to the frequency distributions of the protein scores, using a cut-off score of ≥10 (protein score 0–10 = negative/low expression, and 10–100 = positive expression).

### Cell Viability Assay

Cell viability was evaluated by the colorimetric WST-1 assay as previously described (Hassan et al., 2017; Hassan et al., 2018). Briefly, equal numbers of esophageal cancer cells were plated in a 96-well plate in regular growth medium containing 10% FBS. After 16 h, the medium was replaced with 2% FBS containing phenol red free RPMI1640 medium and the cells were treated with flavopiridol, NPT and BMS-754807 alone or in combinations. After 72 h, 10 μl WST-1 reagent was added in each well followed by additional incubation for 2 h. The absorbance at 450 nm was measured using a microplate reader.

### Western Blot Analysis

Western blot analyses were determined as described by us previously (Hassan et al., 2013a; Hassan et al., 2013b; Hassan et al., 2018). Protein lysates were prepared by treating sub-confluent cells with flavopiridol, NPT, BMS-754807 alone or in combinations, and lysed after 16 h for Western blotting. Polyacrylamide gel electrophoresis was used to separate equal amounts of protein samples, which were then transferred to nitrocellulose membranes. After blocking for 1 h at room temperature membranes were incubated overnight at 4°C with the following primary antibodies: cleaved caspase-3, cleaved poly (ADP-ribose) polymerase-1 (c-PARP), c-Myc, GAPDH ( all from Cell Signaling Technology, Danvers, MA); Mcl-1 (from Enzo Life Sciences, Farmingdale, NY). Blots were incubated with the corresponding HRP-conjugated secondary antibodies (Pierce Biotechnologies, Santa Cruz, CA) for 1 h at room temperature. Specific bands were detected using the enhanced chemiluminescence reagent (ECL, Perkin Elmer Life Sciences, Boston, MA).

### qRT-PCR

Total RNA was extracted from esophageal cancer cells using the TriZol standard RNA extraction (Thermo Fisher Scientific) protocol according to the manufacturers’ instructions. cDNA was synthesized from 1 µg of total RNA in a final volume of 20 µl with random primers under standard conditions using SuperScript III Reverse Transcriptase (Thermo Fisher Scientific). c-Myc transcript was quantified using PCR SYBR Green assays from BioRad. The expression of GAPDH was used to normalize the results. All reactions were run in triplicate and data were analyzed using the comparative cycle threshold (CT) method (Schmittgen and Livak, 2008). The primers used for real-time PCR were: c-Myc primers (forward: 5′-TGA​GGA​GAC​ACC​GCC​CAC-3′; and reverse: 5′-CAA​CAT​CGA​TTT​CTT​CCT​CAT​CTT​C-3′); GAPDH primers (forward: 5′-CCA​CAT​CGC​TCA​GAC​ACC​AT-3′; and reverse 5′-GTA​AAC​CAT​GTA​GTT​GAG​GTC-3′).

### Subcutaneous Tumor Xenografts

All mouse experiments used in this study were carried out in accordance with the standards and guidelines of the Institutional Animal Care and Use Committee (IACUC) at the University of Notre Dame (IACUC protocol # 18-09-4843). Female athymic nude mice (4–6 weeks old) were subcutaneously injected with a panel of esophageal cancer cell lines (5X10^6^). Each athymic nude mice were injected subcutaneously with equal number (5 million) of cells for each cell line. Measurements of subcutaneous tumor size were started when mice had measurable tumors. All mice had measurable tumor 2 weeks after OE19 esophageal adenocarcinoma cell injection. The mice were then randomly grouped (*n* = 5 per group) and treated intraperitoneally as described earlier (Hassan et al., 2017; Hassan et al., 2018) with vehicle (100 µl 0.1% DMSO), flavopiridol (5 mg/kg in 100 µl of 0.1% DMSO, 5 times a week for 2 weeks) (Lee et al., 2014; Pinto et al., 2020), NPT (10 mg/kg in 100 µl of PBS, 2 times a week for 2 weeks) (Hassan et al., 2018) or BMS-754807 (25 mg/kg in 100 µl of PBS, 5 times a week for 2 weeks) (Awasthi et al., 2016) alone or in combinations. The tumor size was measured twice a week for 4 weeks with slide calipers and tumor volume (TV) was calculated as (W^2^XL)/2, where W is width and L is length of the tumor (Awasthi et al., 2012b). Relative tumor volume (RTV) was calculated according to the following formula; RTV = TV_n_/TV_0_ where TV_n_ is the tumor volume at the day of measurement and TV_0_ is the tumor volume on the first day of measurement (Zamai et al., 2003). Mice weight was measured twice a week during the period of the study. At the end of the study mice were euthanized and tumors were removed, weighted, dissected and processed for immunohistochemical analysis.

### Peritoneal-disseminated Animal Survival Model

Animal survival studies were performed using female non-obese diabetic/severe combined immunodeficient (NOD/SCID) mice (4–6 weeks of age) as previously described (Hassan et al., 2017; Hassan et al., 2018). Briefly, the mice were injected intraperitoneally with OE19 EAC (10×10^6^) cells and 2 weeks after tumor cell injection, mice were randomized (*n* = 5 per group) to receive vehicle (100 µl 0.1% DMSO), flavopiridol (5 mg/kg in 100 µl of 0.1% DMSO, 5 times a week for 2 weeks), NPT (10 mg/kg in 100 µl of PBS, 2 times a week for 2 weeks) (Hassan et al., 2018) or BMS-754807 (25 mg/kg in 100 µl of PBS, 5 times a week for 2 weeks) (Awasthi et al., 2016) alone or in combinations. Animal survival was evaluated from the first day of treatment until death (Hassan et al., 2017).

### Immunofluorescence Analysis

Immunofluorescence was performed on histological sections of 4% paraformaldehyde-fixed OE19 tumor xenografts as described before (Hassan et al., 2018; Hassan et al., 2019). Briefly, paraffin embedded tissue blocks were cut into 5 µm tissue sections, deparaffinized and rehydrated. The tissue sections were incubated with a 1:200 dilution of the Ki67 antibody (ab15580, Abcam, Cambridge, MA) and the cleaved caspase-3 antibody (#9661, Cell Signaling Technology, Danvers, MA), followed by incubation with a 1:200 dilution of an anti-rabbit-Cy3 secondary antibody (Jackson ImmunoResearch Laboratories, West Grove, PA). Slides were mounted using a mounting solution containing 4ʹ,6-diamidino-2-phenylindole (DAPI) (Invitrogen, Carlsbad, CA). Fluorescence microscopy was used to detect fluorescent signals. The Intratumoral proliferative and apoptotic index were determined by calculating the Ki67 and cleaved caspase 3 positive cells from five different high-power fields (HPF) in a blinded manner in each group.

### Statistical Analysis

*In vitro* cell proliferation, proliferative and apoptotic index data were expressed as mean ± standard deviation. Statistical analysis was performed by ANOVA for multiple group comparison and Student’s *t*-test for the individual group comparison. The comparison of animal survival time between different groups was done by using the log-rank test (Hassan et al., 2018) using GraphPad Prism 7.0 Software (GraphPad Software, San Diego, CA). The comparison of the relative tumor volume (RTV) between treatment groups was done by first normalizing the RTV values at day 14 by the mean RTV value of the corresponding group at day 0, and then applying the two-sample *t*-test, implemented in the “t.test” R function. *p* < 0.05 was considered statistically significant.

## Results

### c-Myc Overexpression in Human EC Cell Lines and Tissues

**FIGURE 1:**
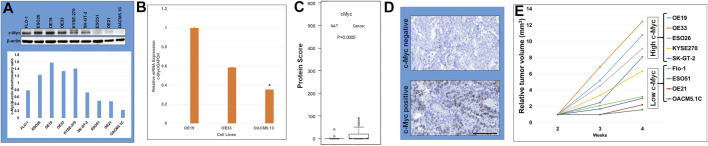
c-Myc overexpression in human esophageal cancer (EC) cell lines and tissues. **(A)** Western blot analysis showing c-Myc expression in EC cell lines. GAPDH serves as loading control. c-Myc over-expression was frequent in this panel of EC cell lines. Densitometric analysis of Western blot showing c-Myc expression the highest in OE19 and the lowest in OACM5.1C. **(B)** c-Myc mRNA expression comparable to protein expression. It is high in OE19/OE33 and low in OACM5.1C. **(C)** There is significantly (*p* < 0.05) higher c-Myc expression in esophageal cancer (Cancer) compared to normal adjacent tissue (NAT) as determined by tissue microarray. **(D)** Immunohistochemistry showing high c-Myc expression in c-Myc positive esophageal cancer compared to c-Myc negative normal esophageal tissue. **(E)** c-Myc overexpression correlated with accelerated esophageal cancer subcutaneous xenograft tumor growth. Five million cells of each cell line were injected subcutaneously in each athymic nude mice.

Using a panel of human esophageal cancer (EC) cell lines, including both esophageal adenocarcinoma (EAC) cell lines (OE19, OE33, ESO26, SK-GT-2, Flo-1, ESO51, and OACM5.1C) and esophageal squamous cell carcinoma (ESCC) cell lines (KYSE270 and OE21), we observed strong expression of c-Myc protein in most cancer lines (Figure 1A). Highest expression of c-Myc protein was observed in OE19, and the lowest expression was observed in OACM5.1C (Figure 1A) which was comparable to c-Myc mRNA expression (Figure 1B). There was a significant (*p* < 0.05) difference of c-Myc expression by TMA between esophageal cancer and normal mucosal esophageal tissue (Figure 1C). Protein score of c-Myc IHC ranged from 0 to 100, with the median value of 44 for esophageal cancer and 6 for normal esophageal tissue. Most of normal esophageal tissues (>90%) had a protein score <10 (negative c-Myc expression, Figure 1D) whereas most of the esophageal cancer tissues (>90%) had a protein score >10 (positive c-Myc expression, Figure 1D). Of the 77 esophageal cancer patients, 49 cases had a protein score >40, considered as IHC c-Myc over-expression. No statistically significant difference of esophageal cancer c-Myc expression was found between different groups of sex, age, tumor location, tumor differentiation, lymph node invasion, and distant metastasis (data not shown).

### Effect of c-Myc Overexpression of Human EC Tumor Growth

C-Myc overexpression correlated with accelerated esophageal cancer subcutaneous xenograft tumor growth (Figure 1E). High c-Myc expressing esophageal cancer cells (OE19, OE33, ESO26, KYSE270, and SK-GT-2) showed enhanced relative tumor volumes compared to low c-Myc expressing esophageal cancer cells (Flo-1, ESO51, OE21, and OACM5.1C) (Figure 1E). OE19 with the highest c-Myc expression showed the highest accelerated esophageal cancer subcutaneous xenograft tumor growth compared to OACM5.1C with the lowest c-Myc expression. In addition, OE19 showed the shortest median animal survival period (45 days), whereas OACM5.1C showed the longest median animal survival period (>120 days) in a peritoneal disseminated metastatic survival model of NOD/SCID mice (Hassan et al., 2017).

### Effect of c-Myc Overexpression on Flavopiridol Induced Human EC Cell Growth Inhibition

**FIGURE 2:**
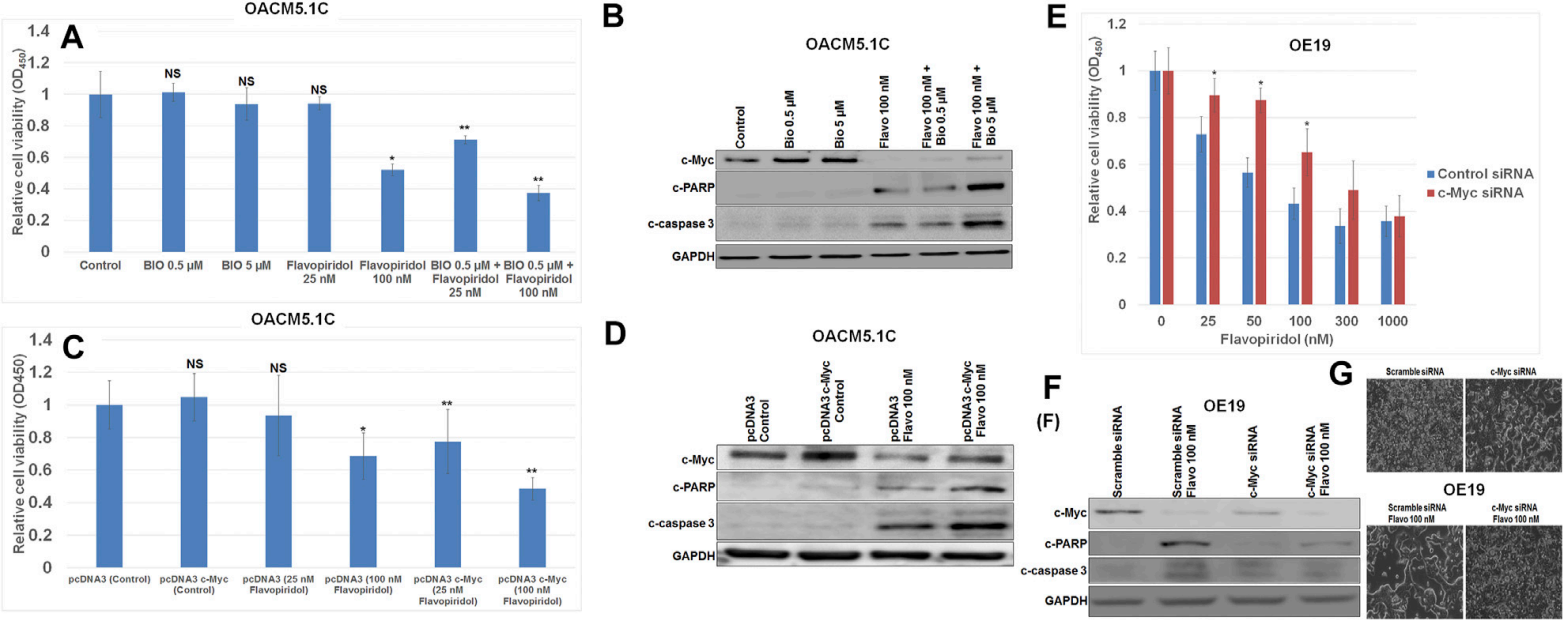
c-Myc overexpression enhanced and c-Myc knockdown diminished flavopiridol induced EC cell death**. (A,B)** Elevated c-Myc expressions either chemically by 6-Bromoindirubin-3ˊ-oxime (BIO) or **(C,D)** genetically by pcDNA3 c-Myc made OACM5.1C cells more sensitive to flavopiridol induced cell death as determined by (A and C) WST-1 cell proliferation assay or by **(B,D)** Western blot analysis compared to those of parent OACM5.1C cells. **(E,F)** Knockdown of c-Myc expressions by c-Myc siRNA made OE19 cells less sensitive to flavopiridol induced cell death as determined by **(E)** WST-1 cell proliferation assay or by **(F)** Western blot analysis compared to those of parent OE19 cells. **(G)** Microscopic picture showing attenuated antiproliferative effect of flavopiridol (Flavo 100 nM) in c-Myc siRNA OE19 cells after knockdown of c-Myc by c-Myc siRNA. In WST-1 assays **(A,C,E)** data are the mean ± SDE of six determinations. In figure **(A)** and **(C)** * represents flavopiridol treatments are significantly different from the control, ** represents BIO + Flavo treatments are significantly different from the corresponding BIO or Flavo treatments, whereas in figure **(E)** * represents 25, 50 and 100 nM flavopiridol treatments after c-Myc siRNA transfection are significantly different from the corresponding concentrations of flavopiridol treatments after control siRNA transfection. In Western blots **(B,D,F)** OACM5.1C cells were treated with BIO, Flavo, BIO + Flavo combinations, also transfected with either pcDNA3 or pcDNA3 c-Myc or c-Myc siRNA followed by treatments with 100 nM Flavo. Total cell extracts were then analyzed by Western blots with antibodies to c-Myc, cleaved PARP (c-PARP), cleaved caspase-3 (c-caspase 3) and GAPDH. Data are representative of three independent experiments with similar results.

C-Myc expression was enhanced by the Wnt signaling activator (6-bromoindirubin-3ˊ -oxime (BIO), a glycogen synthase kinase (GSK)-3β inhibitor that activates c-Myc expression (Meijer et al., 2003; Rottmann et al., 2005) and by plasmid pcDNA3-cMyc in OACM5.1 C esophageal cancer cells which have the lowest c-Myc expression among the tested cell lines. We chose 0.5 and 5 μM of BIO because at these doses BIO significantly increased c-Myc expression without having any significant effects on cell viability/apoptosis in OACM5.1C cells. Interestingly, OACM5.1 C esophageal cancer cells with elevated c-Myc expression by either BIO or pcDNA3-cMyc were preferentially more sensitive to induction of apoptosis by the CDK inhibitor flavopiridol compared to the parent OACM5.1C esophageal cancer cells with lower c-Myc expression (Figures 2A–D). In our experiment flavopiridol (100 nM) decreased c-Myc expression both in OACM5.1C and OE19 cells. In cell viability assays (Figures 2A,C,E) we have used different doses (25–1,000 nM) of flavopiridol to see the dose dependent effect of flavopiridol on these cells. We then used only one dose of flavopiridol (100 nM) which is around IC50 dose in the Western blots (Figures 2B,D,F). c-Myc expression in OACM5.1C cells drastically increased susceptibility to flavopiridol-induced cell growth inhibition as determined by WST assay (Figures 2A,C). Western blot analysis confirmed a greater increase in expression of apoptosis markers cleaved PARP and cleaved caspase 3 after flavopiridol treatment in c-Myc overexpressed OACM5.1C cells (Figures 2B,D). We didn’t see any difference in our results between PBS and DMSO solvent controls in all our experiments when we kept our DMSO concentrations below 0.1%.

### Effect of c-Myc Knockdown on Flavopiridol Induced Human EC Cell Growth Inhibition

Knockdown of c-Myc in high c-Myc expressing OE19 esophageal cancer cells was efficiently achieved by c-Myc siRNA (Niehus et al., 2019) and confirmed by Western blot analysis (Figure 2F). Interestingly, OE19 esophageal cancer cells with knockdown c-Myc expression were preferentially more resistant to induction of apoptosis by the CDK inhibition flavopiridol compared to the parent OE19 esophageal cancer cells with higher c-Myc expression (Figures 2E,F). Decreased c-Myc expression by c-Myc siRNA in OE19 cells significantly decreased susceptibility to flavopiridol-induced cell growth inhibition as determined by WST assay (Figure 2E). Similarly, Western blot analysis confirmed a lesser increase in expression of apoptosis markers cleaved PARP and cleaved caspase 3 after flavopiridol treatment in c-Myc knock-downed OE19 cells (Figure 2F). Finally we showed the microscopic picture (Figure 2G) of attenuated antiproliferative effect of 100 nM flavopiridol with c-Myc siRNA in OE19 cells. With c-Myc siRNA there is slight decreased in cell density compared to scramble siRNA in the absence of flavopiridol (cell density comparison between upper left and upper right square field in Figure 2G). 100 nM of flavopiridol decreased the cell density in scramble siRNA Flavo 100 nM, but cell density increased with c-Myc siRNA in c-Myc siRNA Flavo 100 nM (cell density comparison between lower left and lower right square field in Figure 2G).

### Effect of Flavopiridol on High c-Myc Expressing EC Cell Proliferation and Apoptosis

**FIGURE 3:**
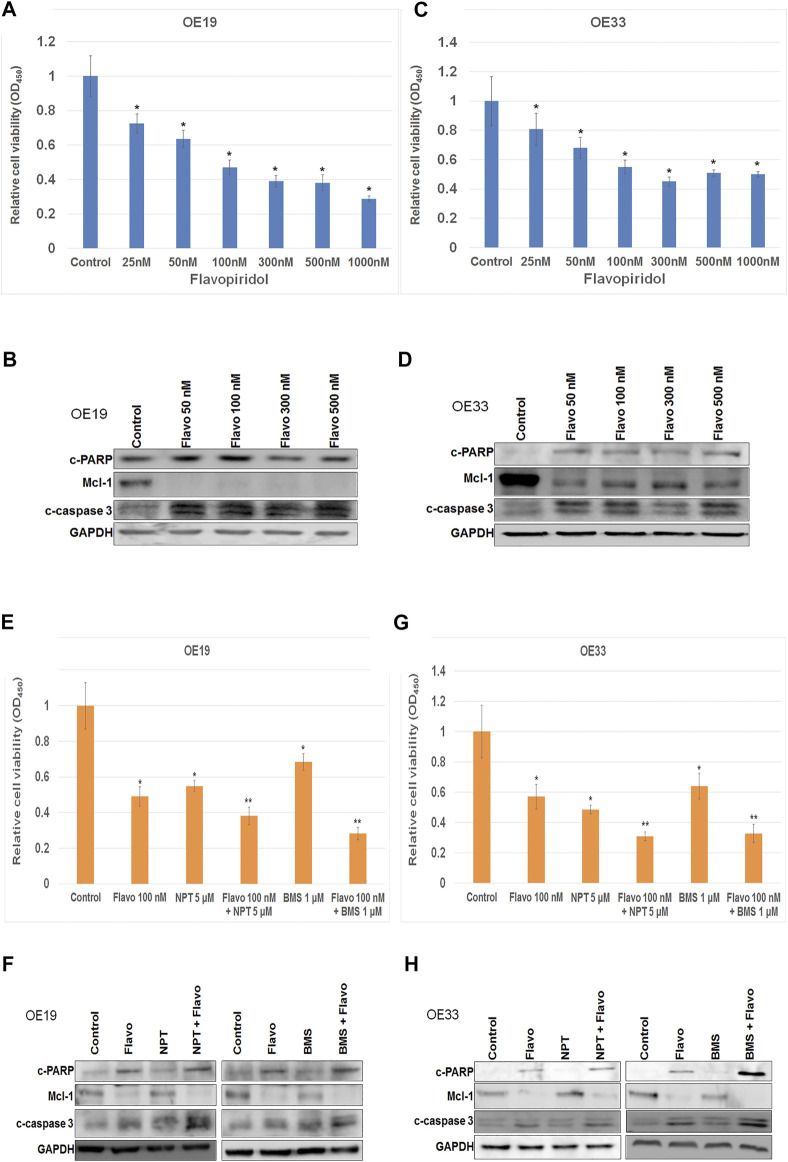
Flavopiridol alone or in combinations induced cell death in c-Myc expressing EC cell. **(A–D)** Nanomolar concentrations of Flavopiridol induced high c-Myc expressing **(A,B)** OE19 and (C and D) OE33 cell death. **(E–H)** Flavopiridol showed enhancement of high c-Myc expressing **(E,F)** OE19 and (G and H) OE33 cell death when it combined with chemotherapeutic agent nanoparticle albumin-bound paclitaxel (NPT) or IGF-1R/IR targeted agent BMS-754807 (BMS). For **(A,C,E,G)** WST-1 assay **(A,C)** OE19 and **(E,G)** OE33 EC cells were plated on 96-well plates and treated with **(A,C)** 25–1,000 nM concentrations of flavopiridol, **(E,G)** 100 nM of flavopiridol alone and in combinations with 5 µM of NPT or 1 µM of BMS. After 72 h, the number of viable cells was calculated by WST-1 assay. Data are the mean ± SDE of six determinations. * represents flavopiridol treatments are significantly different from the control and ** represents Flavo + NPT or Flavo + BMS treatments are significantly different from the monotherapy. In **(B,D,F,H)** western blots cell lysates were analyzed with antibodies to cleaved PARP (c-PARP), Mcl-1, cleaved caspase-3 (c-caspase 3) and GAPDH. Data are representative of three independent experiments with similar results.

*In-vitro* cell viability assays showed that high c-Myc expressing EC cells OE19 and OE33 were very sensitive to flavopiridol induced cell death (Figure 3). Flavopiridol significantly inhibited cell viability of OE19 and OE33 cells in nM concentrations (Figures 3A,C). Reduction in cell viability at 25, 50, 100, 300, 500, and 1,000 nM concentrations of flavopiridol were 27.5, 36.5, 52.8, 61, 61.8, and 71.1%, respectively, in OE19 cells (Figure 3A). Whereas reduction in cell viability in OE33 cells at 25, 50, 100, 300, 500, and 1,000 nM concentrations of flavopiridol were 19.1, 31.8, 45, 54.8, 49, and 50%, respectively, (Figure 3C). Western blot analysis confirmed flavopiridol induced apoptosis by the demonstration of enhanced expression of apoptosis-related markers cleaved caspase-3 and cleaved PARP both in OE19 (Figure 3B) and OE33 (Figure 3D) cells. In addition, flavopiridol drastically reduced anti-apoptotic protein Mcl-1 (Gojo et al., 2002) expression both in OE19 (Figure 3B) and OE33 (Figure 3D) cells.

Flavopiridol in combination with chemotherapy NPT significantly enhanced *in-vitro* cell inhibitory and apoptosis effects both in OE19 and OE33 cells (Figures 3E–H). In addition, flavopiridol in combination with insulin-like growth factor (IGF) targeted therapy BMS-754807 also enhanced *in-vitro* cell inhibitory and cell apoptosis effects both in OE19 and OE33 cells (Figures 3E–H). Furthermore, flavopiridol downregulated Mcl-1 expression, and Mcl-1 expression was completely abolished when flavopiridol was combined with BMS-754807 (Figures 3F,H). In these combination experiments we chose 100 nM of flavopiridol, 5 μM of NPT and 1 μM BMS-754807. In combination experiments very high or very low doses of drugs sometimes failed to show the enhancement of cytotoxic/apoptotic effects. We therefore chose doses around IC25 to IC50 for these drugs. In addition use of these concentrations of flavopiridol, NPT and BMS-754807 have been published previously (Schrump et al., 1998; Carboni et al., 2009; Awasthi et al., 2012a; Awasthi et al., 2016) and all these doses have clinical relevance as determined by pharmacokinetics-pharmacodynamics studies using patients’ blood (Thomas et al., 2002; Desai et al., 2010; Ramaswamy et al., 2012; Chen et al., 2014).

Here we didn’t compare the *in-vitro* antiproliferative/apoptotic effects of flavopiridol between high c-Myc expressing cell line (OE19 or OE33) and low c-Myc expressing cell line (OACM5.1C) as different cell lines may have numerous genetic and epigenetic alterations and such comparisons are not ideal. The OACM5.1C cell line did actually show much lower c-Myc expression compared to OE19 and OE33 (Figure 1A) cell lines. For this part of the study, we therefore chose the high c-Myc expressing cell lines only, and not the lower expressing cell lines.

### Effect of Flavopiridol Mono and Combination Therapies on Human EC Xenograft Tumor Growth

**FIGURE 4:**
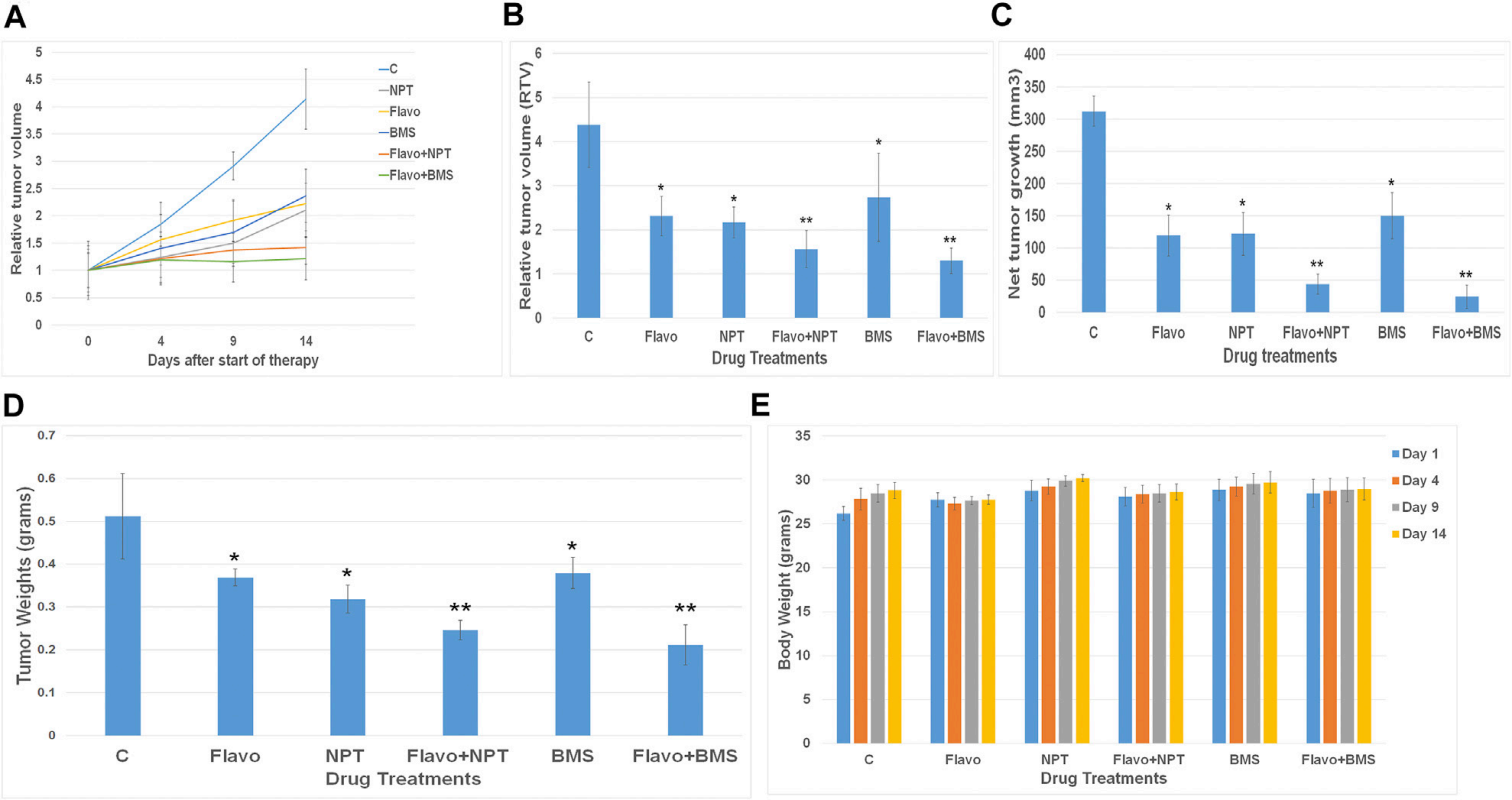
– Xenograft tumor growth inhibition of flavopiridol alone and in combination with chemotherapy or targeted therapy. High c-Myc expressing OE19 cells were subcutaneously injected in nude mice and treated with flavopiridol (Flavo) alone (5 mg/kg in 100 µl of 0.1% DMSO, 5 times a week for 2 weeks) and in combinations with chemotherapeutic agent nanoparticle albumin-bound paclitaxel (NPT) (10 mg/kg in 100 µl of PBS, 2 times a week for 2 weeks) or IGF-1R/IR targeted agent BMS-754807 (BMS) (25 mg/kg in 100 µl of PBS, 5 times a week for 2 weeks) **(A,B)** Relative tumor volume (RTV) was calculated by dividing the tumor volume at any time point by the tumor volume at the start of treatment. **(A)** RTV changes over a period of 4 weeks after injection of cells. **(B)** RTV changes after drug treatments were compared. **(C)** Net growth in tumor size was calculated by subtracting tumor volume on the first treatment day from that of the final day. **(D)** Mean tumor weight was calculated from the final day tumor weights. **(E)** Mouse body weight during the 2-weeks therapy period. Data are representative of mean values ± standard deviation from five mice per group. * indicates significantly different from control **(C)** and ** indicates significantly different single drug therapy.

In OE19 esophageal adenocarcinoma cell-derived subcutaneous xenograft, 2 weeks after tumor cell injection, control mice displayed rapid tumor growth for the next 2 weeks of the therapy period. Flavopiridol monotherapy exhibited a marked tumor regression response to 52.74% compared to control (Figures 4A,B). Flavopiridol with combination chemotherapy NPT showed a significant enhancement effect of tumor regression as tumor size decreased to 35.61% compared to control (Figures 4A,B). BMS-754807 monotherapy also exhibited a significant tumor regression response to 62.41%, and addition of flavopiridol with combination targeted therapy BMS-754807 also showed a significant enhancement effect of tumor regression as tumor size decreased to 29.59% compared to control. (Figure 4A,B). The mean net tumor growth after 14 days was 312.32 ± 23.6 mm^3^ in controls. After flavopiridol monotherapy it significantly decreased to 119.14 ± 31.6 mm^3^, and after NPT monotherapy it significantly decreased to 121.92 ± 33.3 mm^3^, whereas after flavopiridol + NPT combination therapy it was further decreased significantly to 43.77 ± 15.62 mm^3^ (Figure 4C). On the other hand BMS-754807 monotherapy significantly decreased net tumor growth to 149.79 ± 35.8 mm^3^, whereas after flavopiridol + BMS-754807 combination therapy it was further decreased significantly to 24.27 ± 18.27 mm^3^ (Figure 4C). We also observed a significant reduction in xenograft tumor weight by monotherapies (flavopiridol, NPT and BMS-754807), and combination therapies (flavopiridol + NPT and flavopiridol + BMS-754807) further significantly decreased the tumor weight (Figure 4D) without any significant change in mice body weight (Figure 4E). We didn’t see any difference in tumor volumes between PBS (solvents for NPT and BMS-754807) and DMSO (0.1%, solvent for flavopiridol) controls.

### Effect of Flavopiridol Mono and Combination Therapies on Human EC Xenograft Intratumoral Proliferation and Apoptosis

**FIGURE 5:**
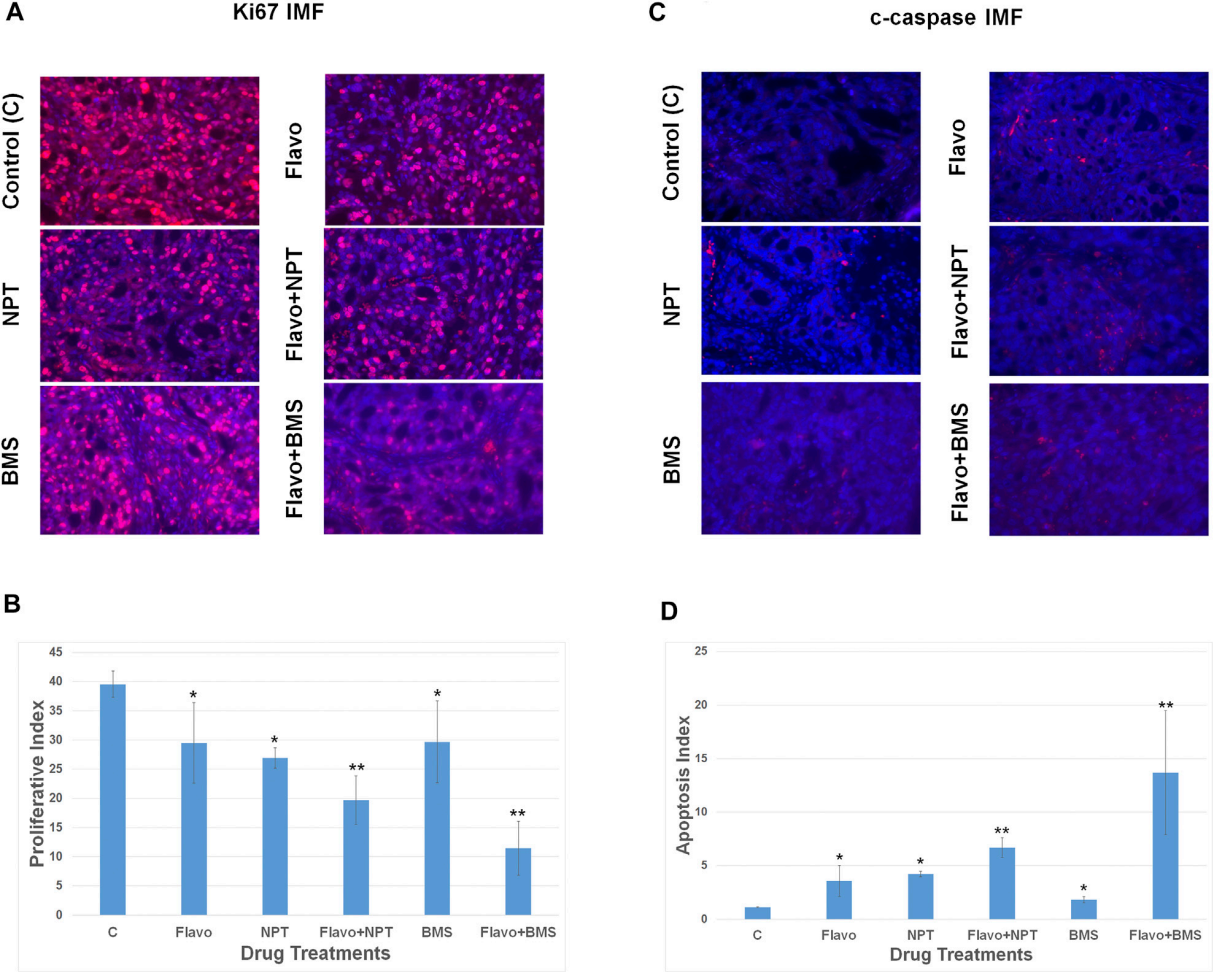
High anti-proliferative and pro-apoptotic in-vivo potency of flavopiridol alone and in combination with chemotherapy or targeted therapy. Nude mice bearing high c-Myc expressing OE19 cell-derived xenografts were treated with flavopiridol (Flavo) alone (5 mg/kg in 100 µl of 0.1% DMSO, 5 times a week for 2 weeks) and in combinations with chemotherapeutic agent nanoparticle albumin-bound paclitaxel (NPT) (10 mg/kg in 100 µl of PBS, 2 times a week for 2 weeks) or IGF-1R/IR targeted agent BMS-754807 (BMS) (25 mg/kg in 100 µl of PBS, 5 times a week for 2 weeks). At completion of treatment, tumors were dissected and proceed for immunofluorescence (IMF). **(A,B)** Intratumoral proliferation was measured by immunostaining tissue sections for Ki67 nuclear antigen (Ki67 IMF). Ki67-postive cells were counted in five different high power fields and plotted as bar graph. **(C,D)** Intratumoral apoptosis was measured by staining tumor tissue sections for cleaved caspase 3 (c-caspase IMF). Cleaved caspase 3-positive cells were counted in five different high power fields and plotted as bar graph. Data are expressed as the mean ± standard deviation. * indicates significantly different from control **(C)** and ** indicates significantly different single drug therapy.

Ki67 staining of OE19 xenografts exhibited significant attenuation of the proliferation of cancer cells by 25.38% with flavopiridol monotherapy compared with that of vehicle-treated control (Figures 5A,B). A combination of chemotherapy NPT or targeted therapy BMS-754807 with flavopiridol were more efficient in attenuation of the cell proliferation than that of monotherapies (Figures 5A,B). Similarly, cleaved caspase-3 staining to determine cancer cell apoptosis in OE19 xenografts exhibited significantly higher apoptosis with flavopiridol monotherapy compared with that of vehicle-treated control (Figures 5C,D). A combination of chemotherapy NPT or targeted therapy BMS-754807 with flavopiridol was more efficient in enhancing apoptosis than that of monotherapies alone (Figures 5C,D).

### Effect of Flavopiridol Mono and Combination Therapies on Animal Survival Harboring Human EC Xenograft

**FIGURE 6:**
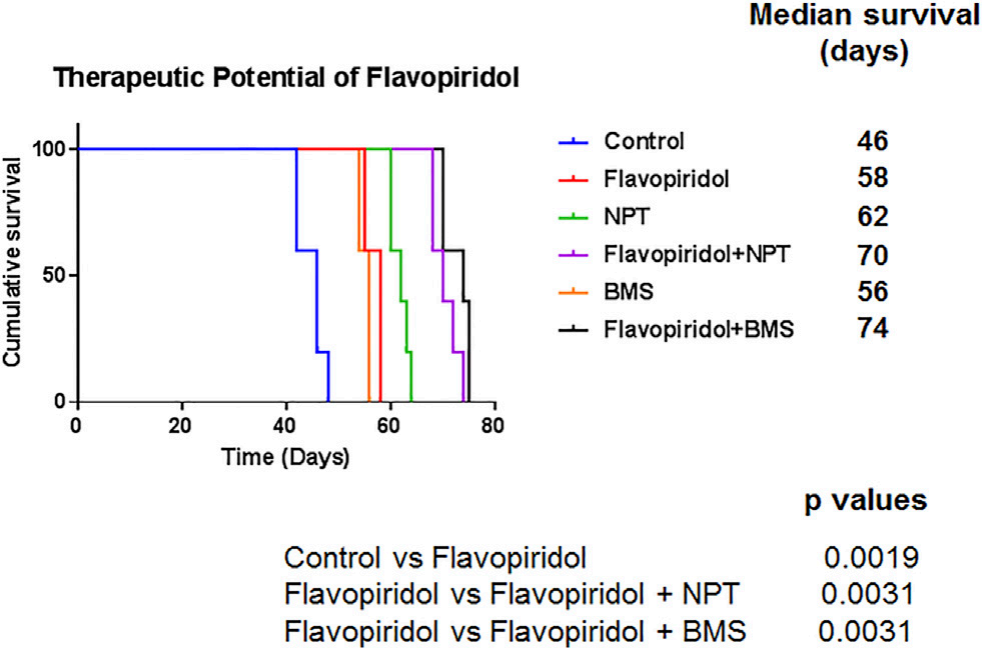
Improvement in animal survival by flavopiridol alone and by addition of chemotherapy or targeted therapy to flavopiridol. Kaplan-Meier survival curve for NOD/SCID mice injected with high c-Myc expressing OE19 cells and treated with flavopiridol alone (5 mg/kg in 100 µl of 0.1% DMSO, 5 times a week for 2 weeks) and in combinations with chemotherapeutic agent nanoparticle albumin-bound paclitaxel (NPT) (10 mg/kg in 100 µl of PBS, 2 times a week for 2 weeks) or IGF-1R/IR targeted agent BMS-754807 (BMS) (25 mg/kg in 100 µl of PBS, 5 times a week for 2 weeks) for 2 weeks. The curve represents the animal survival time from the beginning of therapy. Statistical group differences in survival time were calculated using the log-rank test (GraphPad Prism 7.0).

In the peritoneal disseminated xenograft survival model, the median survival of NOD/SCID mice was 46 days in the control group. Median survival of mice was increased by flavopiridol monotherapy treatment to 58 days (*p* = 0.0019; flavopiridol vs. controls). The combination of chemotherapy NPT or targeted therapy BMS-754807 with flavopiridol significantly increased mouse survival, with the median survival extended to 70 days (*p* = 0.0031; flavopiridol vs flavopiridol + NPT) and 74 days (*p* = 0.0031 flavopiridol vs. flavopiridol + BMS), respectively, (Figure 6).

## Discussion

The proto-oncogene c-Myc encodes a transcription factor that is essential to trigger selective gene expression to promote cancer cell growth and proliferation (Dang, 2012). c-Myc is frequently dysregulated in many human malignancies including esophageal cancer (Tselepis et al., 2003; Li J. et al., 2017; Kalkat et al., 2017; Li et al., 2019; Arman and Möröy, 2020). Although there have been reports of frequent c-Myc amplification in esophageal cancer (von rahden et al., 2006), reports of c-Myc protein expression in esophageal cancer is scarce especially in esophageal adenocarcinoma. In this study, we have demonstrated frequent overexpression of c-Myc protein in a panel of human esophageal cancer cell lines. We also observed higher c-Myc expression in both ESCC and EAC. c-Myc is linked to cellular proliferation and mitogenic growth factor activation in normal cells (Dang, 1999; Eilers and Eisenman, 2008; Dang, 2012; Bretones et al., 2015). In cancer cells, due to their high c-Myc expression, cellular proliferation is no longer dependent on growth-factor induced stimulation (Miller et al., 2012; Dang, 2013). Thus elevated c-Myc expression, as we observed in esophageal cancer cells and tissues, can lead to uncontrolled proliferation of esophageal cancer cells leading to accelerated esophageal cancer growth. Although we have observed accelerated esophageal cancer xenograft tumor growth in high c-Myc expressing esophageal cancer cell lines in this study, we didn’t see any significant association of c-Myc overexpression with patients’ overall survival. Surprisingly, Kaplan-Meier analysis revealed that overall survival tended to be longer in esophageal cancer patients that were c-Myc positive compared with those who were c-Myc negative, but this was not statistically significant (*p* = 0.08; data not shown). In this study, c-Myc expression was identified as an important diagnostic marker for esophageal cancer, but failed to show any prognostic significance. But this study has the limitations of using a small cohort of patients. Furthermore, most of the patients included in this study received adjuvant therapy.

We then explored the role of targeting c-Myc by *in-vitro* studies. Strategies to target c-Myc directly have not been yet achieved and thus multiple pharmacological approaches to indirectly target c-Myc have been exploited (Chen et al., 2018). Synthetic lethal interaction with c-Myc overexpression has been observed with various targets including CDK (Yang et al., 2010; Horiuchi et al., 2012). Flavopiridol is a CDK inhibitor can have antiproliferative/proapoptotic effects in different cell lines through cell cycle arrest and by reducing c-Myc expression (Cobanoglu et al., 2016; Gokce et al., 2016; Dogan turacli et al., 2019). c-Myc overexpression has been reported in many cancer types including esophageal cancer (Tselepis et al., 2003; Dang, 2012; Jung et al., 2017; Wang et al., 2019) as we reported herein, and inhibiting CDK function using small molecule inhibitors (Senderowicz, 2003; Goel et al., 2020) sensitizing c-Myc overexpressing esophageal cancer cells to induce apoptosis can also be exploited. In this study, we have demonstrated the efficacy of flavopiridol, a CDK inhibitor in high c-Myc expressing esophageal cancer cells. We have demonstrated significant reduction in cell proliferation and an increase in apoptosis with nanomolar concentrations. Flavopiridol also demonstrated a dose-dependent antiproliferative effect in high c-Myc expressing OE19 and OE33 esophageal adenocarcinoma cells.

C-Myc expression was manipulated pharmacologically and genetically. We used a Wnt signaling activator (6-bromoindirubin-3ˊ -oxime (BIO) (Zhao et al., 2019) and pcDNA3-cMyc (Ricci et al., 2004) to enhance c-Myc expression in OACM5.1C cells which showed lowest c-Myc expression in our panel of EC cell lines tested. BIO is a novel small molecule inhibitor of glycogen synthase kinase 3 beta (GSK3β) and activator of the wnt/β-catenin pathway (Zhao et al., 2019). Activation of the wnt/β-catenin pathway can upregulate c-Myc expression in various cells (Li et al., 2012; Zhang et al., 2012). In addition, BIO has been shown to influence cell proliferation and stemness in various cells, including cancer cells (Chon et al., 2015; Liu et al., 2017). In this study, we identified BIO as a strong upregulator of c-Myc expression without any significant effect on cell proliferation or apoptosis in OACM5.1C esophageal cancer cells. c-Myc overexpression either by BIO or pcDNA3-cMyc sensitized OACM5.1C cells to CDK inhibitor flavopiridol-induced reduction of cell proliferation and induction of apoptosis. On the other hand, c-Myc expression was depleted by c-Myc siRNAs (Niehus et al., 2019) in high c-Myc expressing esophageal cancer cells. Interestingly, esophageal cancer cells with lowered c-Myc expression were preferentially less sensitive to reduction of cell proliferation or induction of apoptosis by the CDK inhibitor flavopiridol compared to esophageal cancer cells with higher c-Myc expression. This indicates that flavopiridol may be a very effective death inducer of high c-Myc expressing esophageal cancer cells.

Novel combination therapy combining two or more therapeutic agents is a keystone of esophageal cancer therapy (Wiedmann and Mössner, 2013). Nanoparticle albumin bound paclitaxel (NPT) has superior antitumor activity over paclitaxel against esophageal adenocarcinoma (Hassan et al., 2018). NPT is well known for its potent anti-mitotic effect due to its inhibitory effect on cancer cell proliferation, its induction effect on apoptosis and its depletion effect on tumor associated stroma (Awasthi et al., 2013; Hassan et al., 2018). Toxicity associated with combination therapy will be significantly less if different pathways can be targeted (Bayat mokhtari et al., 2017). Combination therapy can enhance the single therapy effect, and therefore a lower therapeutic dosage of each individual drug is required (Bayat mokhtari et al., 2017). Thus it is possible that different mechanisms of actions of flavopiridol and NPT may produce the desired anticancer combination effects with less toxicity. In this study, combining flavopiridol with NPT enhanced reduction of flavopiridol induced *in vitro* cell proliferation and induction of apoptosis in high c-Myc expressing esophageal adenocarcinoma cells. Use of flavopiridol in combination with molecular targeted therapies is very promising and has been used in others cancers, but not in esophageal cancer (Wu et al., 2002). The CDK4/6 inhibitor palbociclib has been used previously with a IGF-1R inhibitor and sensitized its effect (Murakami et al., 2016). Targeting selected cell cycle regulators individually, or in combination with IGF-1R inhibitors may thus provide an efficacious treatment approach that needs to be further validated. There was a report of combining flavopiridol and HER-2 targeting Trastuzumab that resulted in enhanced apoptosis and decreased EGFR expression in breast cancer (Wu et al., 2002). In this study, we have targeted the type I insulin-like growth factor receptor 1R/IR (IGF-1R) with the small molecule inhibitor BMS-754807 (Awasthi et al., 2012a) in high c-Myc expressing OE19 esophageal cancer cells, as OE19 showed very strong expression of IGF-1R (data not shown). Combining flavopiridol with BMS-754807 significantly enhanced reduction in cell proliferation and induction of apoptosis compared to single agent treatment. Flavopiridol with BMS-754807 combination treatment had even higher antiproliferative/proapoptotic effects than flavopiridol with NPT combination treatment in OE 19 cells. These data indicate that flavopiridol is an efficacious inhibitor of high c-Myc expressing esophageal cancer cell growth that may act in combination with other established anticancer agents. In this study, we have not chosen to compare the effects of flavopiridol on esophageal cancer tumor growth between high c-Myc and low c-Myc expressing esophageal cancer cell lines as different cell lines have many genetic and epigenetic alterations and such comparison may not be ideal. However, such comparison of the effects of flavopiridol on esophageal cancer tumor growth will be done in future in the same esophageal cancer cell line with either silencing or overexpressing c-Myc in tetracycline inducible expression systems. Immunoblot analysis revealed that the *in-vitro* antiproliferative effect of flavopiridol mono and combination treatments corresponded with the increased expression of proapoptotic markers cleaved caspase-3, and cleaved PARP. These results indicate the potential enhancement of the apoptotic effect of flavopiridol in combination with NPT and the targeted therapy BMS-754807. It has been shown by other studies that various short-lived proteins like Mcl-1 and c-Myc are downstream targets of CDK (Bettayeb et al., 2010; Hydbring et al., 2017; Tong et al., 2017). Because Mcl-1 is an important antiapoptotic protein in the cell death pathway (Morel et al., 2009), targeting CDK to decrease Mcl-1 expression or enhance Mcl-1 degradation is a rational option for treatment of various cancers including esophageal cancer (Bettayeb et al., 2010; Tong et al., 2017). In this study, flavopiridol drastically decreased expression of the antiapoptotic protein Mcl-1 in OE19 and OE33 esophageal cancer cells. More interestingly, downregulation of Mcl-1 expression was further enhanced when flavopiridol was specifically combined with BMS-754807. Thus removal of growth factors like IGF signaling at any stage of the cell cycle prompts Mcl-1 downregulation which may be due to alteration of specific target genes. Trastuzumab and flavopiridol combination has been found to be synergistic with the transcriptional inhibition of the epidermal growth factor receptor (EGFR) (Nahta et al., 2003). Thus, it could be possible that Mcl-1 is a candidate target of flavopiridol-BMS-754807 combination through inhibition of transcription which warrants further investigation. Flavopiridol also decreased expression of c-Myc in EC cells as expression of c-Myc in cancer cells is closely associated with cell cycle progression and cell growth (Dang, 1999).

In this study, the *in-vitro* decrease in proliferation and increase in apoptosis by flavopiridol correlated with its *in-vivo* antitumor effects. Subcutaneous, intraperitoneal and orthotropic mouse xenograft models have been used for testing *in-vivo* anticancer drugs (Hassan et al., 2017; Hassan and von Holzen, 2018). The FDA approved, clinically utilized CDK inhibitor flavopiridol has shown *in-vivo* antitumor activities in multiple tumor models, especially in hematological malignancies, but not in esophageal cancer (Wiernik, 2016). In this study, flavopiridol alone or in combination with the chemotherapeutic agent NPT or in combination with the targeted agent BMS-754807 significantly inhibited high c-Myc expressing OE19 esophageal cancer subcutaneous xenograft tumor growth without significantly changing average mice body weight. In addition, analysis of intratumoral proliferation and apoptosis also appears to be correlated with xenograft tumor growth inhibition. The OE19 cell line was established from an esophagogastric junction (EGJ) tumor which is rising in incidence in the western world (Chai and Jamal, 2012), and EGJ tumors frequently metastasize to the peritoneal cavity (Strandby et al., 2020). In this study, we observed that flavopiridol monotherapy or combination therapies exhibited significant survival benefit with significantly enhancing overall mice survival in a OE19 peritoneal disseminated mouse xenograft model that has similarities with clinical EGJ cancers (Hassan et al., 2017). Thus high c-Myc expression may therefore represent a valid marker of therapeutic activity of the CDK inhibitor flavopiridol in esophageal adenocarcinoma and warrant clinical validation.

In conclusion, these results support that aggressive esophageal cancer cells with elevated c-Myc expression are an effective targeting option for the CDK inhibitor flavopiridol, and flavopiridol alone or in combination with cytotoxic or targeted agents can be a potential option for high c-Myc expressing esophageal cancer therapy.

## Data Availability

The original contributions presented in the study are included in the article/Supplementary Material, further inquiries can be directed to the corresponding author.

## References

[B1] Arman K. Möröy T. (2020). Crosstalk between MYC and lncRNAs in Hematological Malignancies. Front. Oncol. 10, 579940. 10.3389/fonc.2020.579940 33134177 PMC7579998

[B2] Awasthi N. Zhang C. Ruan W. Schwarz M. A. Schwarz R. E. (2012a). BMS-754807, a Small-Molecule Inhibitor of Insulin-like Growth Factor-1 Receptor/insulin Receptor, Enhances Gemcitabine Response in Pancreatic Cancer. Mol. Cancer Ther. 11, 2644–2653. 10.1158/1535-7163.MCT-12-0447 23047891

[B3] Awasthi N. Zhang C. Ruan W. Schwarz M. A. Schwarz R. E. (2012b). Evaluation of Poly-Mechanistic Antiangiogenic Combinations to Enhance Cytotoxic Therapy Response in Pancreatic Cancer. PLoS One 7, e38477. 10.1371/journal.pone.0038477 22723862 PMC3377661

[B4] Awasthi N. Zhang C. Schwarz A. M. Hinz S. Wang C. Williams N. S. (2013). Comparative Benefits of Nab-Paclitaxel over Gemcitabine or Polysorbate-Based Docetaxel in Experimental Pancreatic Cancer. Carcinogenesis 34, 2361–2369. 10.1093/carcin/bgt227 23803690 PMC4023322

[B5] Awasthi N. Scire E. Monahan S. Grojean M. Zhang E. Schwarz M. A. (2016). Augmentation of Response to Nab-Paclitaxel by Inhibition of Insulin-like Growth Factor (IGF) Signaling in Preclinical Pancreatic Cancer Models. Oncotarget. 10.18632/oncotarget.9029 PMC5216918 27127884

[B6] Bayat mokhtari R. Homayouni T. S. Baluch N. Morgatskaya E. Kumar S. Das B. (2017). Combination Therapy in Combating Cancer. Oncotarget 8, 38022–38043. 10.18632/oncotarget.16723 28410237 PMC5514969

[B7] Bettayeb K. Baunbæk D. Delehouze C. Loaëc N. Hole A. J. Baumli S. (2010). CDK Inhibitors Roscovitine and CR8 Trigger Mcl-1 Down-Regulation and Apoptotic Cell Death in Neuroblastoma Cells. Genes Cancer 1, 369–380. 10.1177/1947601910369817 21779453 PMC3092200

[B8] Bollschweiler E. Hölscher A. H. Schmidt M. Warnecke-Eberz U. (2015). Neoadjuvant Treatment for Advanced Esophageal Cancer: Response Assessment before Surgery and How to Predict Response to Chemoradiation before Starting Treatment. Chin. J. Cancer Res. 27, 221–230. 10.3978/j.issn.1000-9604.2015.04.04 26157318 PMC4490195

[B9] Bretones G. Delgado M. D. León J. (2015). Myc and Cell Cycle Control. Biochim. Biophys. Acta 1849, 506–516. 10.1016/j.bbagrm.2014.03.013 24704206

[B10] Bubendorf L. Nocito A. Moch H. Sauter G. (2001). Tissue Microarray (TMA) Technology: Miniaturized Pathology Archives for High-Throughput *In Situ* Studies. J. Pathol. 195, 72–79. 10.1002/path.893 11568893

[B11] Carboni J. M. Wittman M. Yang Z. Lee F. Greer A. Hurlburt W. (2009). BMS-754807, a Small Molecule Inhibitor of Insulin-like Growth factor-1R/IR. Mol. Cancer Ther. 8, 3341–3349. 10.1158/1535-7163.MCT-09-0499 19996272

[B12] Chai J. Jamal M. M. (2012). Esophageal Malignancy: a Growing Concern. World J. Gastroenterol. 18, 6521–6526. 10.3748/wjg.v18.i45.6521 23236223 PMC3516225

[B13] Chen H. Liu H. Qing G. (2018). Targeting Oncogenic Myc as a Strategy for Cancer Treatment. Signal. Transduct Target. Ther. 3, 5. 10.1038/s41392-018-0008-7 29527331 PMC5837124

[B14] Chen N. Li Y. Ye Y. Palmisano M. Chopra R. Zhou S. (2014). Pharmacokinetics and Pharmacodynamics of Nab-Paclitaxel in Patients with Solid Tumors: Disposition Kinetics and Pharmacology Distinct from Solvent-Based Paclitaxel. J. Clin. Pharmacol. 54, 1097–1107. 10.1002/jcph.304 24719309 PMC4302229

[B15] Chon E. Flanagan B. de Sá Rodrigues L. C. Piskun C. Stein T. J. (2015). 6-Bromoindirubin-3'oxime (BIO) Decreases Proliferation and Migration of Canine Melanoma Cell Lines. Vet. J. 205, 305–312. 10.1016/j.tvjl.2014.07.012 25130776 PMC4312555

[B16] Cobanoglu G. Turacli I. D. Ozkan A. C. Ekmekci A. (2016). Flavopiridol's Antiproliferative Effects in Glioblastoma Multiforme. J. Cancer Res. Ther. 12, 811–817. 10.4103/0973-1482.172132 27461656

[B17] Dang C. V. (2012). MYC on the Path to Cancer. Cell 149, 22–35. 10.1016/j.cell.2012.03.003 22464321 PMC3345192

[B18] Dang C. V. (1999). c-Myc Target Genes Involved in Cell Growth, Apoptosis, and Metabolism. Mol. Cel Biol 19, 1–11. 10.1128/mcb.19.1.1 PMC83860 9858526

[B19] Dang C. V. (2013). MYC, Metabolism, Cell Growth, and Tumorigenesis. Cold Spring Harb Perspect. Med. 3. 10.1101/cshperspect.a014217 PMC3721271 23906881

[B20] Desai J. Solomon B. J. Davis I. D. Lipton L. R. Hicks R. Scott A. M. (2010). Phase I Dose-Escalation Study of Daily BMS-754807, an Oral, Dual IGF-1R/insulin Receptor (IR) Inhibitor in Subjects with Solid Tumors. Jco 28, 3104. 10.1200/jco.2010.28.15_suppl.3104

[B21] Dogan turacli I. Demirtas Korkmaz F. Candar T. Ekmekci A. (2019). Flavopiridol's Effects on Metastasis in KRAS Mutant Lung Adenocarcinoma Cells. J. Cel Biochem 120, 5628–5635. 10.1002/jcb.27846 30317654

[B22] Domper arnaL M. J. Ferrández Arenas Á. Lanas Arbeloa Á. (2015). Esophageal Cancer: Risk Factors, Screening and Endoscopic Treatment in Western and Eastern Countries. World J. Gastroenterol. 21, 7933–7943. 10.3748/wjg.v21.i26.7933 26185366 PMC4499337

[B23] Eilers M. Eisenman R. N. (2008). Myc's Broad Reach. Genes Dev. 22, 2755–2766. 10.1101/gad.1712408 18923074 PMC2751281

[B24] Goel B. Tripathi N. Bhardwaj N. Jain S. K. (2020). Small Molecule CDK Inhibitors for the Therapeutic Management of Cancer. Curr. Top. Med. Chem. 20, 1535–1563. 10.2174/1568026620666200516152756 32416692

[B25] Goga A. Yang D. Tward A. D. Morgan D. O. Bishop J. M. (2007). Inhibition of CDK1 as a Potential Therapy for Tumors Over-expressing MYC. Nat. Med. 13, 820–827. 10.1038/nm1606 17589519

[B26] Gojo I. Zhang B. Fenton R. G. (2002). The Cyclin-dependent Kinase Inhibitor Flavopiridol Induces Apoptosis in Multiple Myeloma Cells through Transcriptional Repression and Down-Regulation of Mcl-1. Clin. Cancer Res. 8, 3527–3538. 12429644

[B27] Gokce O. Dogan Turacli I. Ilke Onen H. Erdem O. Erguven Kayaa E. Ekmekci A. (2016). Flavopiridol Induces Apoptosis via Mitochondrial Pathway in B16F10 Murine Melanoma Cells and a Subcutaneous Melanoma Tumor Model. Acta Dermatovenerol Croat. 24, 2–12. 27149123

[B28] Hassan M. S. Awasthi N. Li J. Schwarz M. A. Schwarz R. E. von Holzen U. (2017). A Novel Intraperitoneal Metastatic Xenograft Mouse Model for Survival Outcome Assessment of Esophageal Adenocarcinoma. PLoS One 12, e0171824. 10.1371/journal.pone.0171824 28225784 PMC5321464

[B29] Hassan M. S. Awasthi N. Li J. Williams F. Schwarz M. A. Schwarz R. E. (2018). Superior Therapeutic Efficacy of Nanoparticle Albumin Bound Paclitaxel over Cremophor-Bound Paclitaxel in Experimental Esophageal Adenocarcinoma. Transl Oncol. 11, 426–435. 10.1016/j.tranon.2018.01.022 29475139 PMC5884213

[B30] Hassan M. S. Von Holzen U. (2018). Animal Model: Xenograft Mouse Models in Esophageal Adenocarcinoma. Methods Mol. Biol. 1756, 151–164. 10.1007/978-1-4939-7734-5_14 29600368

[B31] Hassan M. S. Williams F. Awasthi N. Schwarz M. A. Schwarz R. E. Li J. (2019). Combination Effect of Lapatinib with Foretinib in HER2 and MET Co-activated Experimental Esophageal Adenocarcinoma. Sci. Rep. 9, 17608. 10.1038/s41598-019-54129-7 31772236 PMC6879590

[B32] Hassan S. Karpova Y. Baiz D. Yancey D. Pullikuth A. Flores A. (2013a). Behavioral Stress Accelerates Prostate Cancer Development in Mice. J. Clin. Invest. 123, 874–886. 10.1172/JCI63324 23348742 PMC3561807

[B33] Hassan S. Karpova Y. Flores A. D'Agostino R. JR. Kulik G. (2013b). Surgical Stress Delays Prostate Involution in Mice. PLoS One 8, e78175. 10.1371/journal.pone.0078175 24223137 PMC3819334

[B34] Horiuchi D. Kusdra L. Huskey N. E. Chandriani S. Lenburg M. E. Gonzalez-Angulo A. M. (2012). MYC Pathway Activation in Triple-Negative Breast Cancer Is Synthetic Lethal with CDK Inhibition. J. Exp. Med. 209, 679–696. 10.1084/jem.20111512 22430491 PMC3328367

[B35] Huppi K. Pitt J. J. Wahlberg B. M. Caplen N. J. (2012). The 8q24 Gene Desert: an Oasis of Non-coding Transcriptional Activity. Front. Genet. 3, 69. 10.3389/fgene.2012.00069 22558003 PMC3339310

[B36] Hydbring P. Castell A. Larsson L. G. (2017). MYC Modulation Around the CDK2/p27/SKP2 Axis, 8. Genes (Basel). 10.3390/genes8070174 PMC5541307 28665315

[B37] Jung M. Russell A. J. Liu B. George J. Liu P. Y. Liu T. (2017). A Myc Activity Signature Predicts Poor Clinical Outcomes in Myc-Associated Cancers. Cancer Res. 77, 971–981. 10.1158/0008-5472.CAN-15-2906 27923830

[B38] Kalkat M. DE Melo J. Hickman K. A. Lourenco C. Redel C. Resetca D. (2017). MYC Deregulation in Primary Human Cancers, 8. Genes (Basel). 10.3390/genes8060151 PMC5485515 28587062

[B39] Lee H. G. Baek J. W. Shin S. J. Kwon S. H. Cha S. D. Park W. J. (2014). Antitumor Effects of Flavopiridol on Human Uterine Leiomyoma *In Vitro* and in a Xenograft Model. Reprod. Sci. 21, 1153–1160. 10.1177/1933719114525266 24572052 PMC4212340

[B40] Li E. Liu L. Li F. Luo L. Zhao S. Wang J. (2017a). PSCA Promotes Prostate Cancer Proliferation and Cell-Cycle Progression by Up-Regulating C-Myc. Prostate 77, 1563–1572. 10.1002/pros.23432 28971496

[B41] Li J. Liang Y. Lv H. Meng H. Xiong G. Guan X. (2017b). miR-26a and miR-26b Inhibit Esophageal Squamous Cancer Cell Proliferation through Suppression of C-MYC Pathway. Gene 625, 1–9. 10.1016/j.gene.2017.05.001 28476684

[B42] Li W. Zhang L. Guo B. Deng J. Wu S. Li F. (2019). Exosomal FMR1-AS1 Facilitates Maintaining Cancer Stem-like Cell Dynamic Equilibrium via TLR7/NFκB/c-Myc Signaling in Female Esophageal Carcinoma. Mol. Cancer 18, 22. 10.1186/s12943-019-0949-7 30736860 PMC6367809

[B43] Li Y. Gao Q. Yin G. Ding X. Hao J. (2012). WNT/β-catenin-signaling Pathway Stimulates the Proliferation of Cultured Adult Human Sertoli Cells via Upregulation of C-Myc Expression. Reprod. Sci. 19, 1232–1240. 10.1177/1933719112447126 22872488

[B44] Liu K. Li J. Wu X. Chen M. Luo F. Li J. (2017). GSK-3β Inhibitor 6-Bromo-Indirubin-3'-Oxime Promotes Both Adhesive Activity and Drug Resistance in Colorectal Cancer Cells. Int. J. Oncol. 51, 1821–1830. 10.3892/ijo.2017.4163 29039496

[B45] Mawhinney M. R. Glasgow R. E. (2012). Current Treatment Options for the Management of Esophageal Cancer. Cancer Manag. Res. 4, 367–377. 10.2147/CMAR.S27593 23152702 PMC3496368

[B46] Mccarty K. S. JR. Miller L. S. Cox E. B. Konrath J. Mccarty K. S. (1985). Estrogen Receptor Analyses. Correlation of Biochemical and Immunohistochemical Methods Using Monoclonal Antireceptor Antibodies. Arch. Pathol. Lab. Med. 109, 716–721. 3893381

[B47] Mcmahon S. B. (2014). MYC and the Control of Apoptosis. Cold Spring Harb Perspect. Med. 4, a014407. 10.1101/cshperspect.a014407 24985130 PMC4066641

[B48] Meijer L. Skaltsounis A. L. Magiatis P. Polychronopoulos P. Knockaert M. Leost M. (2003). GSK-3-selective Inhibitors Derived from Tyrian Purple Indirubins. Chem. Biol. 10, 1255–1266. 10.1016/j.chembiol.2003.11.010 14700633

[B49] Miller D. M. Thomas S. D. Islam A. Muench D. Sedoris K. (2012). c-Myc and Cancer Metabolism. Clin. Cancer Res. 18, 5546–5553. 10.1158/1078-0432.CCR-12-0977 23071356 PMC3505847

[B50] Morel C. Carlson S. M. White F. M. Davis R. J. (2009). Mcl-1 Integrates the Opposing Actions of Signaling Pathways that Mediate Survival and Apoptosis. Mol. Cel Biol 29, 3845–3852. 10.1128/MCB.00279-09 PMC2704749 19433446

[B51] Muenst S. Schaerli A. R. Gao F. Däster S. Trella E. Droeser R. A. (2014). Expression of Programmed Death Ligand 1 (PD-L1) Is Associated with Poor Prognosis in Human Breast Cancer. Breast Cancer Res. Treat. 146, 15–24. 10.1007/s10549-014-2988-5 24842267 PMC4180714

[B52] MurakamI T. Singh A. S. Kiyuna T. Dry S. M. Li Y. James A. W. (2016). Effective Molecular Targeting of CDK4/6 and IGF-1R in a Rare FUS-ERG Fusion CDKN2A-Deletion Doxorubicin-Resistant Ewing's Sarcoma Patient-Derived Orthotopic Xenograft (PDOX) Nude-Mouse Model. Oncotarget 7, 47556–47564. 10.18632/oncotarget.9879 27286459 PMC5216960

[B53] Nahta R. Trent S. Yang C. Schmidt E. V. (2003). Epidermal Growth Factor Receptor Expression Is a Candidate Target of the Synergistic Combination of Trastuzumab and Flavopiridol in Breast Cancer. Cancer Res. 63, 3626–3631. 12839951

[B54] Niehus S. E. Allister A. B. Hoffmann A. Wiehlmann L. Tamura T. Tran D. D. H. (2019). Myc/Max Dependent Intronic Long Antisense Noncoding RNA, EVA1A-AS, Suppresses the Expression of Myc/Max Dependent Anti-proliferating Gene EVA1A in a U2 Dependent Manner. Sci. Rep. 9, 17319. 10.1038/s41598-019-53944-2 31754186 PMC6872820

[B55] Pandilla R. Kotapalli V. Gowrishankar S. Chigurupati M. Patnaik S. Uppin S. (2013). Distinct Genetic Aberrations in Oesophageal Adeno and Squamous Carcinoma. Eur. J. Clin. Invest. 43, 1233–1239. 10.1111/eci.12163 24102414

[B56] Pinto N. Prokopec S. D. Ghasemi F. Meens J. Ruicci K. M. Khan I. M. (2020). Flavopiridol Causes Cell Cycle Inhibition and Demonstrates Anti-cancer Activity in Anaplastic Thyroid Cancer Models. PLoS One 15, e0239315. 10.1371/journal.pone.0239315 32970704 PMC7514001

[B57] Ramaswamy B. Phelps M. A. Baiocchi R. Bekaii-Saab T. Ni W. Lai J. P. (2012). A Dose-Finding, Pharmacokinetic and Pharmacodynamic Study of a Novel Schedule of Flavopiridol in Patients with Advanced Solid Tumors. Invest. New Drugs 30, 629–638. 10.1007/s10637-010-9563-7 20938713 PMC3486515

[B58] Ricci M. S. Jin Z. Dews M. Yu D. Thomas-Tikhonenko A. Dicker D. T. (2004). Direct Repression of FLIP Expression by C-Myc Is a Major Determinant of TRAIL Sensitivity. Mol. Cel Biol 24, 8541–8555. 10.1128/MCB.24.19.8541-8555.2004 PMC516765 15367674

[B59] Rottmann S. Wang Y. Nasoff M. Deveraux Q. L. Quon K. C. (2005). A TRAIL Receptor-dependent Synthetic Lethal Relationship between MYC Activation and GSK3beta/FBW7 Loss of Function. Proc. Natl. Acad. Sci. U S A. 102, 15195–15200. 10.1073/pnas.0505114102 16210249 PMC1257707

[B60] Rubenstein J. H. Shaheen N. J. (2015). Epidemiology, Diagnosis, and Management of Esophageal Adenocarcinoma. Gastroenterology 149, 302–e1. 10.1053/j.gastro.2015.04.053 25957861 PMC4516638

[B61] Russo P. Arzani D. Trombino S. Falugi C. (2003). c-Myc Down-Regulation Induces Apoptosis in Human Cancer Cell Lines Exposed to RPR-115135 (C31H29NO4), a Non-peptidomimetic Farnesyltransferase Inhibitor. J. Pharmacol. Exp. Ther. 304, 37–47. 10.1124/jpet.102.042952 12490573

[B62] Rustgi A. K. El-serag H. B. (2014). Esophageal Carcinoma, N. Engl. J. Med., 12, 30. 10.1056/nejmra1314530 25539106

[B63] Schmittgen T. D. Livak K. J. (2008). Analyzing Real-Time PCR Data by the Comparative C(T) Method. Nat. Protoc. 3, 1101–1108. 10.1038/nprot.2008.73 18546601

[B64] Schrump D. S. Matthews W. Chen G. A. Mixon A. Altorki N. K. (1998). Flavopiridol Mediates Cell Cycle Arrest and Apoptosis in Esophageal Cancer Cells. Clin. Cancer Res. 4, 2885–2890. 9829756

[B65] Senderowicz A. M. (2003). Small-molecule Cyclin-dependent Kinase Modulators. Oncogene 22, 6609–6620. 10.1038/sj.onc.1206954 14528286

[B66] Siegel R. L. Miller K. D. Jemal A. (2015). Cancer Statistics, 2015. CA Cancer J. Clin. 65, 5–29. 10.3322/caac.21254 25559415

[B67] Stine Z. E. Walton Z. E. Altman B. J. Hsieh A. L. Dang C. V. (2015). MYC, Metabolism, and Cancer. Cancer Discov. 5, 1024–1039. 10.1158/2159-8290.CD-15-0507 26382145 PMC4592441

[B68] Strandby R. B. Svendsen L. B. Ambrus R. Rostved A. A. Hasselby J. P. Achiam M. P. (2020). The Incidence of Free Peritoneal Tumor Cells before and after Neoadjuvant Chemotherapy in Gastroesophageal Junction Cancer. J. Cytol. 37, 40–45. 10.4103/JOC.JOC_164_18 31942097 PMC6947737

[B69] Thomas J. P. Tutsch K. D. Cleary J. F. Bailey H. H. Arzoomanian R. Alberti D. (2002). Phase I Clinical and Pharmacokinetic Trial of the Cyclin-dependent Kinase Inhibitor Flavopiridol. Cancer Chemother. Pharmacol. 50, 465–472. 10.1007/s00280-002-0527-2 12451473

[B70] Tong Z. Chatterjee D. Deng D. Veeranki O. Mejia A. Ajani J. A. (2017). Antitumor Effects of Cyclin Dependent Kinase 9 Inhibition in Esophageal Adenocarcinoma. Oncotarget 8, 28696–28710. 10.18632/oncotarget.15645 28404924 PMC5438684

[B71] Torre L. A. Bray F. Siegel R. L. Ferlay J. Lortet-Tieulent J. Jemal A. (2015). Global Cancer Statistics, 2012. CA Cancer J. Clin. 65, 87–108. 10.3322/caac.21262 25651787

[B72] Tselepis C. Morris C. D. Wakelin D. Hardy R. Perry I. Luong Q. T. (2003). Upregulation of the Oncogene C-Myc in Barrett's Adenocarcinoma: Induction of C-Myc by Acidified Bile Acid *In Vitro* . Gut 52, 174–180. 10.1136/gut.52.2.174 12524396 PMC1774961

[B73] Von rahden B. H. Stein H. J. Pühringer-Oppermann F. Sarbia M. (2006). c-Myc Amplification Is Frequent in Esophageal Adenocarcinoma and Correlated with the Upregulation of VEGF-A Expression. Neoplasia 8, 702–707. 10.1593/neo.06277 16984727 PMC1584294

[B74] Wang T. Cai B. Ding M. Su Z. Liu Y. Shen L. (2019). c-Myc Overexpression Promotes Oral Cancer Cell Proliferation and Migration by Enhancing Glutaminase and Glutamine Synthetase Activity. Am. J. Med. Sci. 358, 235–242. 10.1016/j.amjms.2019.05.014 31324362

[B75] Wiedmann M. W. Mössner J. (2013). New and Emerging Combination Therapies for Esophageal Cancer. Cancer Manag. Res. 5, 133–146. 10.2147/CMAR.S32199 23869177 PMC3706320

[B76] Wiernik P. H. (2016). Alvocidib (Flavopiridol) for the Treatment of Chronic Lymphocytic Leukemia. Expert Opin. Investig. Drugs 25, 729–734. 10.1517/13543784.2016.1169273 26998706

[B77] Wu K. Wang C. D'Amico M. Lee R. J. Albanese C. Pestell R. G. (2002). Flavopiridol and Trastuzumab Synergistically Inhibit Proliferation of Breast Cancer Cells: Association with Selective Cooperative Inhibition of Cyclin D1-dependent Kinase and Akt Signaling Pathways. Mol. Cancer Ther. 1, 695–706. 12479366

[B78] Yang D. Liu H. Goga A. Kim S. Yuneva M. Bishop J. M. (2010). Therapeutic Potential of a Synthetic Lethal Interaction between the MYC Proto-Oncogene and Inhibition of aurora-B Kinase. Proc. Natl. Acad. Sci. U S A. 107, 13836–13841. 10.1073/pnas.1008366107 20643922 PMC2922232

[B79] Zamai M. Vandeven M. Farao M. Gratton E. Ghiglieri A. Castelli M. G. (2003). Camptothecin Poly[n-(2-Hydroxypropyl) Methacrylamide] Copolymers in Antitopoisomerase-I Tumor Therapy: Intratumor Release and Antitumor Efficacy. Mol. Cancer Ther. 2, 29–40. 12533670

[B80] Zhang S. Li Y. Wu Y. Shi K. Bing L. Hao J. (2012). Wnt/β-catenin Signaling Pathway Upregulates C-Myc Expression to Promote Cell Proliferation of P19 Teratocarcinoma Cells. Anat. Rec. (Hoboken) 295, 2104–2113. 10.1002/ar.22592 22976998

[B81] Zhang Y. (2013). Epidemiology of Esophageal Cancer. World J. Gastroenterol. 19, 5598–5606. 10.3748/wjg.v19.i34.5598 24039351 PMC3769895

[B82] Zhao X. E. Yang Z. Gao Z. Ge J. Wei Q. Ma B. (2019). 6-Bromoindirubin-3'-oxime Promotes Osteogenic Differentiation of Canine BMSCs through Inhibition of GSK3β Activity and Activation of the Wnt/β-Catenin Signaling Pathway. Acad. Bras Cienc 91, e20180459. 10.1590/0001-3765201920180459 30916158

